# P02 Uncovering the role of NAD(P)H quinone oxidoreductases in *Pseudomonas aeruginosa*: new avenues for antibiotic potentiators

**DOI:** 10.1093/jacamr/dlaf230.009

**Published:** 2025-12-04

**Authors:** C Stylianou, W Saleh, B Ivanov, S Holland, V Crescente, E Polycarpou, E Sim, N J Saunders, A Ryan

**Affiliations:** Department of Applied Sciences, Northumbria University, Newcastle upon Tyne, NE1 8ST, UK; College of Health, Medicine and Life Sciences, Brunel University, London, UB8 3PH, UK; School of Life Sciences, Pharmacy and Chemistry, Kingston University, Kingston upon Thames KT1 2EE, UK; School of Life Sciences, Pharmacy and Chemistry, Kingston University, Kingston upon Thames KT1 2EE, UK; School of Life Sciences, Pharmacy and Chemistry, Kingston University, Kingston upon Thames KT1 2EE, UK; School of Life Sciences, Pharmacy and Chemistry, Kingston University, Kingston upon Thames KT1 2EE, UK; School of Life Sciences, Pharmacy and Chemistry, Kingston University, Kingston upon Thames KT1 2EE, UK; School of Life Sciences, Pharmacy and Chemistry, Kingston University, Kingston upon Thames KT1 2EE, UK; Department of Applied Sciences, Northumbria University, Newcastle upon Tyne, NE1 8ST, UK; School of Life Sciences, Pharmacy and Chemistry, Kingston University, Kingston upon Thames KT1 2EE, UK

## Abstract

**Background:**

Antimicrobial resistance (AMR) is a critical global health challenge, with Gram-negative pathogens such as *Pseudomonas aeruginosa* posing a major threat due to their multidrug resistance and clinical prevalence in hospital-acquired infections*. P. aeruginosa* demonstrates resistance to several antibiotics, including aminoglycosides, fluoroquinolones and β-lactams. While traditional resistance mechanisms have been well characterized, NAD(P)H quinone oxidoreductases (NQOs) have been implicated in antibiotic resistance through their role in protecting cells from oxidative stress triggered by certain antibiotics.

**Methods:**

This study investigates the role of NQOs in *P. aeruginosa* by generating a series of single and multi-gene knock-out strains using a suicide plasmid system, with complementation via Gateway cloning.

**Results:**

Antibiotic susceptibility testing revealed that NQO-deficient strains exhibit increased resistance to aminoglycosides but heightened susceptibility to fluoroquinolones. These findings suggest a differential role for NQOs in modulating antibiotic efficacy depending on the antibiotic class. Additionally, NQO knock-outs displayed altered virulence-related phenotypes, including changes in swarming motility, biofilm formation and pyocyanin production.

**Conclusions:**

Ongoing work is focused on characterizing NQO expression in response to antibiotic treatment and determining their contribution to oxidative stress regulation. Collectively, these findings support the role of NQOs in *P. aeruginosa* resistance and virulence and propose them as promising targets for the development of antibiotic potentiators- compounds that enhance antibiotic effectiveness by disrupting bacterial stress responses. This research highlights a novel strategy to combat AMR and improve therapeutic outcomes against high-risk pathogens.

